# Primary Epithelioid Angiosarcoma of the Colon With Peritoneal Carcinomatosis

**DOI:** 10.7759/cureus.36709

**Published:** 2023-03-26

**Authors:** Quan Do, Zainab A Farooqui, Dipendra Parajuli

**Affiliations:** 1 Internal Medicine, University of Louisville School of Medicine, Louisville, USA

**Keywords:** sarcoma, epithelioid angiosarcoma, colorectal cancer, carcinomatosis peritoneal, metastatic angiosarcoma

## Abstract

Angiosarcoma is a rare mesenchymal tissue neoplasm, typically involving lymphatic or vascular endothelial cells. The tumor can arise anywhere in the body, though it is most often found as cutaneous lesions in the head and neck region. Due to its rarity, a diagnosis can sometimes be missed, especially when the sarcoma involves an uncommon site like the gastrointestinal tract. In this case, we describe a male patient who was found to have primary epithelioid angiosarcoma of the colon. Initial biopsies with immunohistochemistry staining were weakly positive for anti-cytokeratin (CAM 5.2) and negative for SRY-Box transcription factor 10 (SOX-10) and B-cell-specific activator protein (PAX-5). He was misdiagnosed as having poorly differentiated carcinoma as a result. However, a more in-depth look at the colon specimen after tumor resection revealed CD-31 and factor VIII positivity, which established the diagnosis of epithelioid angiosarcoma of the colon. This case suggests the use of rare histopathology markers as part of the workup for colonic lesions to confirm the diagnosis, especially when tissue biopsy is limited.

## Introduction

Angiosarcoma is a rare malignancy involving lymphatic or vascular endothelial cells that represents 1% of all soft tissue sarcomas [1]. It has an estimated incidence of about one to two cases per million [2]. These highly aggressive tumors can arise anywhere in the body, although the most common sites are cutaneous lesions of the head and neck and occasional involvement of the spleen, liver, bone, and other visceral organs. Involvement of the gastrointestinal tract is very rare and accounts for less than 1% of all colorectal malignancies [3]. Treatment for this tumor is very difficult and often has poor results, particularly in metastatic disease [1]. In this case report, we discuss a rare case of metastatic colonic angiosarcoma with peritoneal carcinomatosis.

## Case presentation

**Figure 1:**
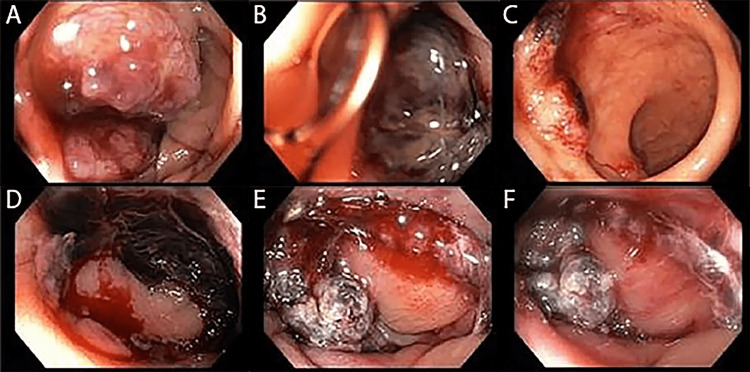
Endoscopic gross appearance: (A), (B), and (C): multiple oozing masses from the patient’s rectum to 40 cm up his left colon prior to surgical resection. (D) and (E): bleeding from a new metastatic mass near the anastomosis site a month after surgical resection; and (F): after epinephrine injection.

A male patient in his 70s with a history of diverticulitis presented after two months of worsening rectal bleeding. He was recently transfused with three units of blood due to the bleeding. His routine screening colonoscopy two years prior was negative for malignancy. On examination, the patient’s vital signs were stable, and the physical exam was only pertinent for bright red blood per rectum. His laboratory tests showed anemia with a hemoglobin of 6.6 g/dL, platelets of 324 ×109/L, reticulocyte count of 3.6%, and normal coagulation factors. An urgent colonoscopy was performed, and he was found to have multiple oozing masses from his rectum to 40cm up his left colon (Figure 1A-1C). A biopsy of the left colonic mass revealed colonic mucosa infiltrated by poorly differentiated carcinoma based upon the interpretation of immunostaining for anti-cytokeratin (CAM 5.2) as weakly positive as well as negative immunostaining for caudal-type homeobox 2 (CDX2), SRY-box transcription factor 10 (SOX-10), and B-cell-specific activator protein (PAX-5).

**Figure 2:**
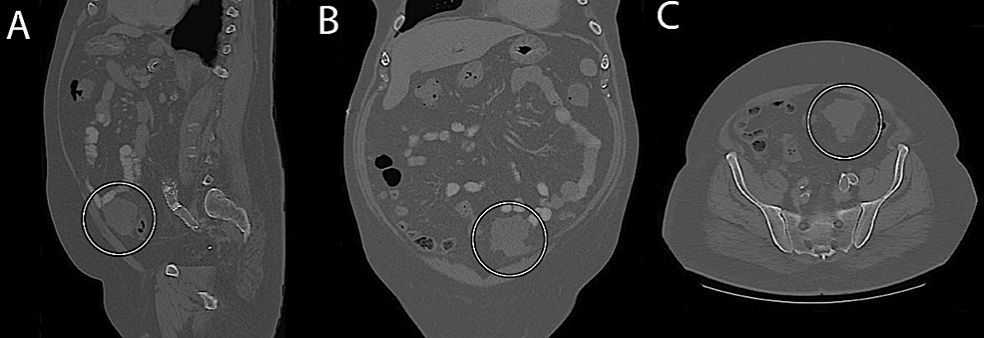
A CT scan prior to surgical resection showed a mesenteric metastatic mass measuring 5.8 cm seen in the anterior lower abdomen (circled in white), along with multiple smaller peritoneal implants throughout the abdomen: (A) sagittal view, (B) coronal view, and (C) axial view.

Computed tomography (CT) of the chest, abdomen, and pelvis revealed an anterior abdominal mass measuring 5.8 cm, consistent with a mesenteric metastasis, along with multiple smaller implants seen throughout the anterior lower abdomen (Figure 2). These CT findings were highly concerning for peritoneal carcinomatosis. The intraluminal oozing masses seen during the colonoscopy were not visible in the CT scan.

**Figure 3:**
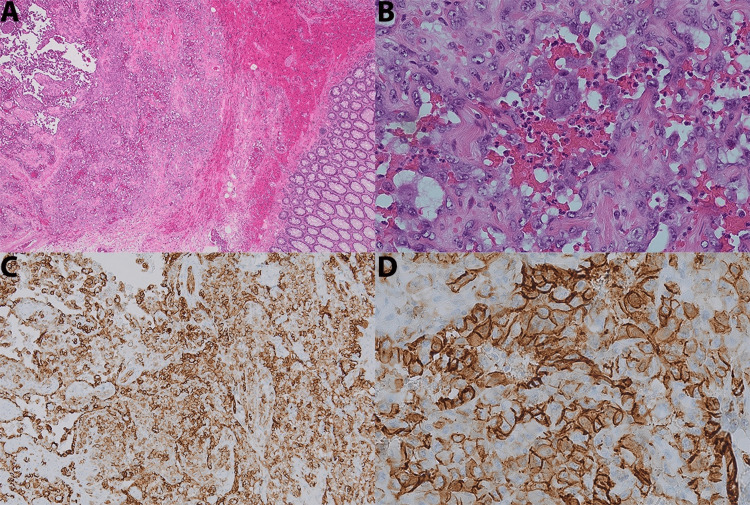
(A) Histopathology showing a tumor involving the colonic submucosa (hematoxylin and eosin (H&E), 40x); (B) tumor with epithelioid morphology with prominent small to medium-sized vessels (H&E, 200x); (C) CD-31 positivity (immunohistochemistry, 40x); and (D) CD-31 positivity (immunohistochemistry, 400x).

Colorectal surgery was consulted, and a biopsy of the peritoneal implants was needed to further guide the treatment decision. Intraoperatively, a few of these peritoneal implants were taken out and sent for a rapid frozen section. The frozen section showed only inflammatory tissue and reactive cells; thus, low anterior resection (LAR) with anastomosis and ileostomy formation was performed. The anterior abdominal mass was also resected. Unfortunately, as additional tissues were submitted for pathology post-surgery, these implants were indeed metastatic lesions and not reactive cells. The tumor on the colon resection specimen was proven to be angiosarcoma, and the correct diagnosis was confirmed as a highly atypical epithelioid angiosarcoma. The pathological specimen was R0, with proximal and distal resection margins free of disease. Findings were notable for tumor invasion through the muscularis propria into the colonic mesentery and peritoneum. The infiltrating neoplasm was immunoreactive for vascular endothelial markers, CD-31, and Factor VIII, further supporting the diagnosis of angiosarcoma (Figure 3). Eight pericolic lymph nodes were dissected and were all negative. Pathologic staging was pT3N0M1.

**Figure 4:**
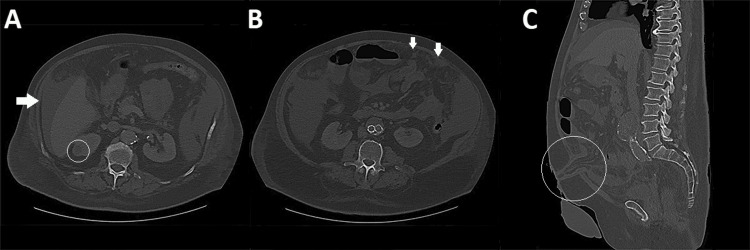
CT scan a month after surgery showing: (A) new large volume ascites (white arrow) and a new right kidney lesion measuring 2.5 cm (white circle); (B) worsening peritoneal implants (white arrows); and (C) an ileostomy (white circle).

After surgery, chemotherapy with liposomal doxorubicin was planned. However, a repeat CT of the chest, abdomen, and pelvis a month after surgery showed worsening nodular infiltration of the omentum and mesentery and a new 2.5 cm right renal mass, complicated by large volume ascites (Figure 4).

The patient opted for palliative chemoradiation with paclitaxel instead. He continued, however, to have symptoms of rectal bleeding. A repeat colonoscopy showed bleeding from a new metastatic mass near the anastomosis site (Figure 1D-1F). Epinephrine was injected in an attempt to stop the bleeding, but there was no improvement. He underwent a mesenteric angiogram with embolization with interventional radiology but showed only minimal improvement. He passed away shortly after, approximately four months after the diagnosis.

## Discussion

Angiosarcomas of the gastrointestinal tract are a very rare and aggressive malignancy that constitute less than 1% of gastrointestinal (GI) malignancies [3]. GI angiosarcomas most commonly occur in the small bowel and stomach; however, colonic involvement has been noted, typically in the sigmoid colon followed by the rectum [4]. Our case supported this finding with the involvement of the rectum and left colon. To our knowledge, there are 33 cases reported of primary colonic angiosarcoma [3]. The typical manifestations of this malignancy include rectal bleeding, perianal pain, weight loss, and diarrhea. These nonspecific symptoms can lead to delayed diagnosis and cause rapid hematogenous spread, contributing to the disease's poor prognosis. In this case, the patient presented with a general complaint of GI bleed, with the only risk factor being a history of diverticulitis.

Although the majority of angiosarcomas are sporadic, chronic inflammation is a known risk factor, along with chemoradiation, toxins, and foreign body exposure [5]. To our knowledge, our patient did not have any exposure to the known risk factors. His routine screening colonoscopy was also completely normal two years prior to the presentation. This could be explained by the rapid and highly aggressive nature of angiosarcoma. These highly vascular tumors typically express endothelial cell markers, which can be confirmed with immunohistochemical (IHC) staining. In this case, the infiltrating malignant neoplasm was immunoreactive for CD-31 and Factor VIII, which confirmed our diagnosis.

It is important to remain cognizant of diseases that can present similarly to angiosarcomas when searching for a diagnosis of this rare malignancy. Our patient was originally diagnosed with poorly differentiated carcinoma based on the immunostaining of a weakly positive CAM 5.2. Epithelioid angiosarcomas can mimic these carcinomas; however, positive stains for CD-31 and factor VIII can help clarify the diagnosis. It is also important to compare the two morphologically, as there have been few cases reporting metastatic carcinomas with positive CD-31 markers [6]. However, this marker has more specificity and sensitivity for the endothelial association. Kaposi's sarcoma must also be differentiated from angiosarcoma due to its similar histological features and IHC staining patterns; however, the patient population is typically associated with HIV and has immunopositivity for CD-117 (KIT) and CD-34 markers. These markers can also be present in epithelioid gastrointestinal stromal tumors (GIST), which are high on the differential when diagnosing angiosarcomas. Furthermore, a literature review found that angiosarcomas may also test positive for CD-117 and CD-34, which should prompt additional testing to stratify the disease [3]. Although rare in the gastrointestinal tract, epithelioid sarcoma is another differential diagnosis that can be ruled out by immunohistochemical markers like CD-31 and the erythroblast transformation-specific-related gene (ERG) [7,8]. Other highly malignant tumors such as metastatic melanoma (SOX-10 marker) and lymphoma (CD-19, PAX-5, terminal deoxynucleotidyl transferase (TdT) markers, etc.) should also be considered in differentials [9,10].

Due to the invasive nature of angiosarcomas, total surgical resection with wide margins is the main treatment of choice. Our patient underwent surgery and was subsequently treated with paclitaxel chemotherapy; however, he succumbed to his disease shortly after diagnosis. Although the role of adjuvant chemotherapy is not well established, taxanes are considered the primary regimen, and cytotoxic drugs have shown benefit in metastatic angiosarcomas [11]. Evidence for improvement with chemotherapy is limited, however, cases have shown resolution of lesions with increased survival up to 38 months with adjuvant paclitaxel [12]. At this time, there is not enough literature to support or oppose treatment with chemotherapy, and surgery remains the only curative option. Unfortunately, the mortality rate for angiosarcomas within 12 months of surgical resection remains high at 62%, with younger patients having an overall better post-surgical prognosis than patients older than 60 years of age [13,14].

## Conclusions

Colonic angiosarcomas are rare invasive malignancies that should not be overlooked in patients with vague abdominal symptoms such as rectal bleeding, abdominal pain, or diarrhea. It is oftentimes misdiagnosed as carcinoma, leading to delayed medical attention and rapid metastatic spread. These tumors are highly aggressive and often respond poorly to chemotherapy, with a high mortality rate after surgical resection. Early detection is imperative and can augment management. Given the poor prognostic nature of colonic angiosarcomas, it is important to make a correct diagnosis and continue to report this rare malignancy for further research into management.
